# Challenges facing the management of pesticide resistance in weeds, diseases and insect pests in European agriculture and the future of effective IPM implementation

**DOI:** 10.1002/ps.70522

**Published:** 2026-01-24

**Authors:** Julian Smith, Bianca Assis Barbosa Martins, Roland Beffa, Linda M Field, Andreas Goertz, Gael Le Goupil, Andreas Mehl, Juergen Langewald, Samuel Martinelli, Caio Vitagliano Santi Rossi, John A Wiles

**Affiliations:** ^1^ 10 The Rise Leavening UK; ^2^ Bayer AG, Division CropScience Frankfurt Germany; ^3^ Senior Scientist Consultant CLI/HRAC Liederbach Germany; ^4^ Rothamsted Research Ltd Harpenden UK; ^5^ Bayer AG, Division CropScience Monheim am Rhein Germany; ^6^ Syngenta Crop Protection Basel Switzerland; ^7^ BASF SE, APR/IL Limburgerhof Germany; ^8^ Bayer Crop Science Saint Louis MI USA; ^9^ Rossi & D Manejo, CLI/HRAC Uberlândia Brazil; ^10^ Corteva Agriscience, Melbourn Science Park Melbourn UK

**Keywords:** pesticide resistance, resistance management, sustainability, stewardship, integrated pest management

## Abstract

In recent times, pesticide resistance has been managed reasonably effectively, either proactively or reactively, by monitoring resistance of pest biotypes and the rotation of products with different modes of action (MoAs). However, increased regulation is dramatically limiting the range of MoAs available to farmers, especially in Europe. Innovation and replenishment with new MoAs from industry cannot keep pace with this loss, leaving the need for pragmatic choices in how to manage pests effectively through all methods available. This is crucial for integrated pest management (IPM) adoption to support sustainable crop production. Here we consider the current situation for insecticides, herbicides and fungicides in Europe and suggest that, despite the emerging IPM options, in many cropping systems, the need for a pesticide component remains essential. As part of efficient IPM or resistance management (RM) strategies, the availability of a range of effective pesticide MoAs will be essential. In addition, for more productive and sustainable agricultural systems, all stakeholders, including the agrochemical industry, farmers/growers, advisory services, the research community and policy/decision makers of Europe should try to improve communication. This will be the only way to ensure the future production of sufficient, high‐quality crops, at a time when there are many threats to food security in Europe. © 2026 The Author(s). *Pest Management Science* published by John Wiley & Sons Ltd on behalf of Society of Chemical Industry.

## Graphical abstract

Opinion on pesticide resistance and management in the European Union (EU) and United Kingdom (UK) is presented cognisant of changes in regulation that impact on mode of action availability and cropping system choice.

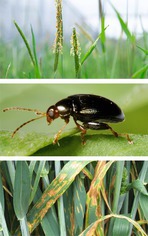

## 1. INTRODUCTION

Food security in Europe depends on having efficient and productive agricultural systems that ensure economic food production, whilst minimising negative effects on biodiversity and the environment more widely. Part of successful crop production is the prevention of losses that result from competing weeds and attack by diseases and insect pests (the Food and Agriculture Organisation of the United Nations (FAO)^1^ have estimated that up to 40% of global crop production is lost due to plant pests). Currently, and especially in field production, pest control is largely achieved using pesticides. However, with the aim of reducing pesticide use, increased emphasis is being placed on integrated pest management (IPM) where pesticides are used alongside other non‐chemical control methods (see later).

Whether pesticides are used as the main method of crop protection, or as part of IPM, it is accepted that when pests are repeatedly exposed to the same mode of action (MoA), Darwin's theory of evolution and selection will prevail. In these situations MoA resistance will become a very real and dynamic risk to crop production. So how do we slow down and keep ahead of the resistance arms race? The Resistance 2024 conference at Rothamsted Research, sponsored by the three Resistance Action Committees (RACs), Herbicide RAC (HRAC), Fungicide RAC (FRAC) and Insecticide RAC (IRAC) provided a unique opportunity for industry, government and academics from around the world to address this question and take a holistic view of the challenges of resistance management (RM). As part of this there was a panel discussion of the current challenges facing crop protection and how best to integrate RM into the wider aspirations of IPM. In this article we consider this from a European agriculture perspective, highlighting the challenges from the point of view of each of the RACs and Rothamsted Research.

## 2. A UNIFIED PERSPECTIVE ON RESISTANCE MANAGEMENT FROM THE RESISTANCE ACTION COMMITTEES

The sections below provide perspectives from IRAC, HRAC, and FRAC and present a unified message that the evolution and selection of resistance in pests (insects, weeds, and diseases) is a critical threat to sustainable agriculture and global food security across the world but specifically across the European Union (EU) and United Kingdom (UK).

During the Resistance 2024 session by the RACs the core argument across all three disciplines was that the foundation of effective RM is maintaining a diverse ‘toolbox’ of chemical pesticide MoAs. The committees stressed that rotating, mixing, or sequencing the use of products, with different MoAs is the most crucial strategy to slow the development of resistance and preserve the long‐term efficacy of chemical controls.

A central and urgent theme is the negative impact of the current regulatory landscape, particularly in Europe. The withdrawal of key active ingredients is shrinking the number of available MoAs, which has several detrimental effects:

- It accelerates resistance to the remaining chemicals by increasing their use and selection pressure.
- It undermines IPM strategies, which often rely on chemical options as a key component.
- It leads to significant economic consequences for farmers, including increased control costs and reduced yields, threatening the viability of certain crops like sugar beet, oilseed rape (OSR), and potatoes.
- It necessitates an increase in Emergency Use Approvals as growers are left without effective solutions for critical pest problems.

Later, the three RAC's provide examples of current challenges and note that the development of new MoAs and the commercialisation of non‐chemical alternatives are not occurring fast enough to fill the gaps left by regulatory withdrawals.

## 3. IRAC PERSPECTIVE

The role of IRAC International is to provide technical leadership and coordination of a global network to support the implementation of insecticide and trait RM programmes by developing and promoting strategies that support sustainable agriculture and improved public health (https://irac-online.org). A key element of effective insecticide RM, as it is for herbicides and fungicides, is the use of alternations, rotations, or sequences of insecticides belonging to different MoA classes, either as single or mixed products. A core principle is that plant protection product users should avoid selecting for insecticide resistance or cross‐resistance by repeated use, within the crop cycle or over successive crops of the same insecticide or related products in the same MoA class. Both European and UK stakeholders may agree that our common interest is providing sustainable insect pest management to support successful harvests and sustain the food supply. Perhaps the question we should ask ourselves is, ‘How are we maintaining/enhancing the toolbox of insect pest management solutions that can be integrated to control the most economically important crop pests in our region?’

There is little doubt that currently the most viable and sustainable solution to insect pest control is continued access to a diversity of pest management tools that can be combined in IPM, where chemical control, with few exceptions, is an essential component.^2^ It is notable that the implementation of IPM is frequently advertised as a strategy in RM to fill the gap for lacking rotation partners. Indeed, IPM by itself is a form of RM. However, because chemical management remains an important component of most contemporary IPM systems, problems with resistance towards the chemicals might not necessarily be solved^3^ if access to different MoAs is not available. The availability of fewer registered compounds often results in increased resistance problems for those remaining on the market. It would be appropriate to consider whether regulatory or political systems may have a disproportionate impact, resulting in the loss of a useful MoA. At the same time, fewer new compounds with a new MoA that can replace old products with resistance issues are being evaluated and/or approved for use in the markets. For instance, the new MoA (IRAC group 36) could be the first new insecticide MoA that will become available for European farmers in 20 years. This may be one reason for the increased ‘Emergency Use Approvals’ that appear necessary at the country level to support growers in managing critical pest problems in important crops.^4^

The other challenge to the IPM toolbox is that for many crop‐pest uses, sufficiently effective or practical non‐chemical solutions are not currently available. Stakeholders, such as researchers and biological companies, are active in trying to enhance the toolbox; however, products or solutions are not being registered or commercialised at a pace that is sufficient to provide either immediate impact or supporting integrated technical field guidance for effective implementation at the grower level across the range of crops required.

While quite advanced IPM tactics are commonly applied in glasshouses and some tree and vine crops, they are more difficult to implement and less established in field crops. Recent evidence has shown that the control of insect vector‐transmitted diseases is very difficult using IPM tactics. For example, in the EU, the lack of systemic products as rotation partners for aphid control in sugar beet is threatening sugar beet production.^5^, ^6^ Consequently, in France, pushed by farmer syndicates and after long public debates, a bill (loi Duplomb, www.legifrance.gouv.fr/jorf/id/JORFTEXT000049026136) was passed by the French parliament, reintroducing certain neonicotinoids for the control of aphids in sugar beet. Similar lack of control options occurs in OSR, where weevil and flea beetle have become resistant to available chemicals, leading to a substantial reduction in acreage.^7^ In a recent study, IPM strategies have been tested and led to a reduction of insecticide applications in OSR; however, at the same time, insecticide applications increased in neighbouring crops such as barley and peas where alternative preventive measures were limited.^7^ IPM strategies can also be fragile. One of the largest threats to established programmes is disruption from the emergence of new, invasive pests or the rise of a new pathosystem, such as diseases in sugar beet and potato transmitted by the beet leafhopper. Management of this pest is still reliant on the use of prophylactic insecticide applications, a practice that is necessary in those situations but viewed as poorly compatible with IPM.^3^

The debate may continue as to what IPM, RM, and insect management will look like in practice in the future. For insect RM (IRM), it would seem critical that we maintain a variety of chemical MoAs in our toolbox that supports effective pest management. At the same time, complementary tools are also essential. In addition to cultural methods, pheromones, and physical barriers already being tested, it will be interesting to see how biological products, or even gene editing, offer additional options. Finally, it will be critical that growers and those managing crops and the agricultural landscape are able to devise and implement insect pest management strategies at the field and farm level that improve IPM (and IRM) and sustain effective crop production under conditions that, due to global warming, are becoming increasingly advantageous for indigenous and invasive pest insects to flourish.^8^, ^9^

## 4. HRAC PERSPECTIVE

Weeds have major impacts on the yield and quality of crops, and control largely relies on using herbicides. However, herbicide resistance is a major threat to sustainable weed management. Overuse and reliance on a single herbicide MoA increases the risk of resistance evolution in weed populations. To mitigate such risk, it is essential to adopt herbicide RM (HRM) strategies, combining agronomy measures with herbicide programmes that incorporate different MoAs to achieve the highest possible diversity in each agronomic system. Unlike insect pest and disease control, HRM for efficiently controlling weeds and mitigating herbicide resistance evolution can combine many agronomic strategies and the use of chemistry. For more than 15 years, HRAC has developed ‘Best Management Practices’, which include crop rotations, soil management (including tillage when necessary and possible), adaptation of seed sowing time, use of cover crops, and alternation of spring and autumn crops. Additionally, strategies like rotating or combining herbicide active ingredients with different MoAs, whether in mixtures or sequences, are critical for maintaining their long‐term efficacy. An additional unique feature of herbicides is the development of diverse herbicide treatment programmes, including the use of the best combinations between pre‐emergence and post‐emergence herbicides. By doing so, farmers can keep their fields clean of weeds and productive while mitigating the evolution of resistance. Banning one MoA or an active ingredient can vastly compromise the efficacy of a given programme and therefore tremendously decrease the use of other herbicides involved in such a programme.

It is important to recognise that herbicide resistance evolution is not only the result of poor practices adopted by farmers but also other factors, including high weed pressures that require herbicide use, the impact of new regulations (especially in Europe) that withdraw key MoAs and chemical classes, and advisories to reduce recommended application rates. The latter is observed, in particular, for the Group 15 herbicides (very long‐chain fatty acid synthesis inhibitors). The impact of withdrawing MoAs can be profound. For example, the consequences of a non‐renewal of glyphosate use approval in the EU have been widely discussed. Finge *et al*.^10^ emphasised the economic implications of a potential glyphosate ban in Europe, projecting impacts ranging from €3 per hectare in silage maize production to as high as €553 per hectare in grapevine cultivation. Similarly, Metcalf *et al*.^11^ modelled the scenario of withdrawing glyphosate and replacing it with cultural control methods, evidencing projected increases in weed abundance, environmental risks, and decreased food production. Overall, the findings emphasise the need for careful consideration of trade‐offs arising in scenarios where glyphosate is removed. This approach should be taken in any proposal aiming to ban a herbicide chemistry or chemical classes representing a given MoA.

It is interesting to learn from outside of Europe, where similar challenges are evident. In Brazil, herbicide‐resistant weeds have resulted in significantly higher control costs and yield losses. For Brazilian soybean fields, managing resistant weed populations can cost up to four times more than in non‐resistant areas, creating a substantial financial burden for farmers. This underscores the critical importance of ensuring access to diverse and effective herbicide active ingredients for sustaining profitability and productivity.

As of 2025, there are over 530 unique herbicide resistance cases reported in more than 270 weed species globally.^12^ In addition, weeds have evolved resistance against most of the 25 known herbicide MoAs currently available. A big concern is the evolution of herbicide resistance based on enhanced metabolism, referred to as non‐target site resistance.^13^

This non‐target site mechanism often confers broad‐spectrum resistance and, for its management along with target‐site resistance, requires the highest diversity possible of both herbicide MoAs and chemical classes. Based on today's genomic knowledge, additional molecular mechanisms are being identified, allowing improved diagnostics, for example, in blackgrass and ryegrass, two grass weeds occurring widely in crops.^14^, ^15^ This is necessary to adapt resistance mitigation strategies.

Managing herbicide resistance requires more than just herbicide stewardship. It requires a holistic approach through HRM. With the continuous evolution of resistance to existing herbicide MoAs and the limited discovery of new MoAs, HRAC is promoting HRM practices worldwide to ensure farmers have access to a broad spectrum of tools. These include awareness of the fullest breadth of available herbicide MoAs alongside best practice management, for example, crop rotation, soil management, and cover crops. As we stand today, weed control is largely achieved at ever‐increasing cost, and it is likely that new and more prevalent resistance will take us to a tipping point of declining productivity.

Policymakers and regulatory bodies must acknowledge the vital role proper use of herbicides plays, not only in mitigating herbicide resistance but also in slowing its progression and spread. Preserving a wide array of effective herbicide options is one pillar of HRM.

## 5. FRAC PERSPECTIVE

As with insect pests and weeds, disease outbreaks caused by plant pathogenic fungi devastate crops and threaten food security. While control can be achieved using fungicides, resistance can evolve and be selected. This resistance can lead to various levels of decreased efficacy of the applied fungicide (or fungicide MoA group) or even to insufficient control of a specific pathogen at the field, regional, or country level. Thus, it is important to anticipate this risk by implementing RM measures and monitoring resistance evolution to best adapt the conditions of fungicide use. The aim is to slow down or avoid resistance evolution and maintain the effectiveness of available fungicides. Incorrect use of a single fungicide MoA increases the risk of resistance evolution and thus, it is of high importance for growers to follow sustainable fungicide RM (FRM) strategies alongside sound agronomic practices. The most efficient strategies include alternating or mixing non‐cross‐resistant active ingredients with different MoAs, respecting the recommended dose rates, and limiting the total number of applications per MoA and season according to the crop, key pathogens, and agricultural conditions.

In this context, it is important to note that effective FRM requires a sufficient number of diverse MoAs and that fungicide resistance is therefore not only exacerbated by insufficient advice or improper product use but, as with herbicide and insecticide resistance, by regulatory withdrawals of effective active ingredients, which reduces the required MoA diversity.

An important example is the recent call from CropLife Europe, together with the Committee of the European Starch Potato Producers' Unions, the European Farmers & European Agri‐Cooperatives, the European Potato Processors' Association, the European Potato Trade Association, and other organisations for an EU Action Plan entitled ‘EU potato production at risk: a call to combat late blight in potatoes’.^16^ Potato late blight (*Phytophthora infestans*) is the most destructive disease of potatoes, recording an annual economic damage in the EU estimated at around €900 million.^17^ Recently, the pathogen population has been developing more complex virulence spectra (i.e., the ability to escape more and more combinations of resistance genes) as well as multiple resistance to single‐site fungicide active ingredients. These developments are reducing the number of effective control tools, potentially to such a low level that current IPM control strategies will no longer be effective.^17^ This is already apparent in the EU, where resistance has been detected to four out of 11 major fungicide MoAs, putting increased pressure on those that remain. This risk is compounded by EU regulations that prevent the use of multisite MoAs. It is anticipated that the emergence of resistance to the remaining MoAs will evolve over the coming seasons. Potato late blight was a scourge for the 2024 growing season, with the potato sector in desperate need of both short‐term and long‐term solutions, including both research and application.^18^ Consequently, according to this call, the following key short‐term needs are:

- Fungicides with five different MoAs must be made available to farmers to ensure effective FRM.
- Currently available fungicides should not be banned or restricted without a proper risk/benefit analysis, and unless a similar effective, sustainable, and affordable alternative, with at least the same level of efficacy, is available on the market.
- There should be a programme of communicating IPM to farmers, including preventive strategies to prevent the breakdown of resistance genes against late blight based on scientific results and models, as well as FRAC guidelines.

Forward‐looking IPM control strategies are feasible by combining the current control strategy with the introduction of more resistant potato varieties, farm management practices against infection, and the availability of fungicides with a range of MoAs for both resistance genes and fungicide active ingredients complementing and protecting each other.

Besides *Phytophthora infestans*, control of other diseases, caused by Oomycetes is also strongly impacted by resistance. For example, grapevine downy mildew (*Plasmopara viticola*) represents the most devastating grapevine disease, and like late blight, its control mostly relies on compounds developed to specifically inhibit Oomycota species. Important active ingredients belong to the chemical groups of phenylamides, benzamides, carboxylic acid amides, quinone inside inhibitors, quinone inside and outside inhibitors (stigmatellin binding mode), carbamates, cyanoacetamide‐oximes, and oxysterol binding protein homologue inhibitors. For sound disease and RM, today's spray schedules usually include a broad diversity of the chemical control options, in line with FRAC use guidelines. However, according to recent FRAC reports, resistance cases have been reported for the majority of effective oomyceticides, and the latest sensitivity monitoring often shows the occurrence of isolates bearing resistance to multiple active ingredients and biochemical MoA. Due to the severe resistance issues and the decreasing toolbox of effective control options, the topic of Oomycetes resistance was specifically addressed during ‘Resistance 2024’, outlining a lack of specific control solutions in certain grapevine and potato growing regions and the need for more innovative chemical options with new MoAs.

In barley, multiple resistance of *Pyrenophora teres* (net blotch) and *Ramularia collo‐cygni* (Ramularia leaf spot) to major fungicide classes represent further examples of increasing disease control issues for growers inside and outside of Europe. The same applies to *Cercospora beticola* (Cercospora leaf spot) in sugar beet across the globe, and to *Corynespora cassiicola* (target spot), a major pathogen of particular relevance in soybean and cotton production in Brazil.

In summary, a broad diversity of fungicide classes with different MoAs is not only crucial for disease control but also mandatory for managing fungicide resistance and limiting the spread of resistant fungal populations once they are present in farmers' fields. Keeping a wide range of effective fungicide solutions should be acknowledged by regulatory bodies and policymakers, as this range forms the basis for sustainable IPM in times of increasing global population and food production challenges, in order to decrease losses of the most important staple crops predicted to increase in a warming world.^19^, ^20^ With the continuous evolution of fungicide resistance and the strongly decreasing number of compounds with new MoAs being introduced into the market per decade, FRAC provides worldwide guidance for best FRM practices of existing solutions to ensure farmers continue their production of high yielding quality crops (www.frac.info).

## 6. ROTHAMSTED RESEARCH PERSPECTIVE

Many of the principles for crop protection and pesticide resistance, raised by the RACs, are central elements of ‘Regenerative Agriculture’ and ‘One‐Health’ as ideologies that seek to frame more inclusive, broader‐thinking and dynamic roadmaps in securing sustainable environment and human health outcomes. Common to both would be a decreased dependence on pesticide use in building ecosystem services that regulate pest risk and damage within acceptable thresholds. In considering such a direction of travel an aspiration for a shared position for farmers, regulators, importers and consumers, formulating best practices becomes very challenging. By example, a farmer's concern is primarily at the field and season level such as decisions on acceptable risks related to whether or when to spray a pesticide. As a farmer producing for the home‐market, there may be a question of acceptable pest‐caused losses (yield and quality) and market value, whereas, if producing for export, zero‐tolerance on a quarantined organism is likely to apply. A regulator, or a trader, may think in different ways, more centred on the larger land scales and longer time periods, for example, a decrease over time of farming inputs; an increase in set‐aside land; an uptick in a stewardship scheme, and so forth. Layered into these choices is the play of consumers and their willingness‐to‐pay for environmental outcomes and the extent that market forces and consumer demand will support those economics without the need for government interventions, that is the UK Department for Environment, Food and Rural Affairs (Defra) Sustainable Farming Incentives (SFIs) or EU equivalent. It is a complex arena with many stakeholders with diverse vantage points and expectations that may be variously aligned or conflicting.

At Resistance 2024, these types of issues were debated, considering tighter regulations linked to higher costs of development, broad spectrum verses more targeted MoAs, and the pragmatic choices made by farmers and supply chains. It is required to square the circle of building ecosystem service (for pest control) by using less pesticides, when even the small, judicious use of a broad MoA pesticide will not discriminate to the benefit of beneficial organisms, and more targeted MoAs are not available. With our current IPM toolbox, it was considered unviable to continue on the current path of reducing pesticide use with a reduced scope of MoAs, expecting farmers to primarily bear the risk.

At Rothamsted, our research is centred on squaring the circle described earlier. Some examples, as highlighted in a recent article,^21^ are given.

### 6.1. Discovery of new active molecules: artificial intelligence‐driven discovery

A recent survey commissioned by Crop Life International indicated that the costs associated with bringing a new active ingredient to the major US and European markets was $301 million (€261 million) and takes over 12 years.^22^ The impact of these high costs and long timelines has been the decline in new MoAs reaching the market. Interestingly the decline is not uniform across fungicide, insecticide and herbicides, with fungicidal MoA introduction notably higher and commercialisation of new herbicidal MoAs lagging. The flat market for new herbicidal MoAs may reflect the effectiveness and market share of glyphosate and glyphosate‐resistant crops.^23^ Against a backdrop of tightening regulatory approval, notably related to off‐target consequences, the prospectus for existing research approaches generating a sufficient pipeline of new pesticide MoAs to effectively manage resistance seems challenged.

New research based on artificial intelligence (AI) and machine learning may present game‐changing potential. ‘AI‐machine learning discovery platforms’ can be directed at *in silico* identification of molecules with enhanced biological activity, ease of manufacturing, reduced toxicity and, importantly, target specificity.^24^ At Rothamsted, AI‐machine learning and *in silico* molecular modelling is fast‐tracking the discovery of novel biocontrol molecules of the future.^25^ Similarly, MoA Technology in Oxford, based in the UK, are exploiting AI to discover new herbicide MoAs.^26^ The subject of new herbicide discovery has recently been reviewed by Duke *et al*.^27^ When mirrored across insect pest and fungi, such discovery of highly targeted MoAs will massively help with enhancing levels of nature‐based control and, as an addition to the toolbox, lessen the pressure driving resistance.

### 6.2. Insect and insecticide resistance: unintended consequences of the neonicotinoid ban in UK

Rothamsted has maintained an ongoing interest in the withdrawal of neonicotinoid seed treatments, where its expertise in assessing farmer practices, land‐change, and sustainability supports farmers, industry and policymakers. In 2013 the EU, as a measure to reverse the decline in pollinators, restricted the use of neonicotinoid seed treatments in crops that flower. For OSR this led to loss of control of cabbage stem flea beetle (*Psylliodes chrysocephala*), which has widespread resistance to pyrethroids (the only other permitted alternative). Consequently, the areas of cultivation to OSR in the UK and across Europe have significantly declined (by almost *c*. 50%), with farmers switching to alternative crops^27^ having their own management practice and impact on biodiversity and sustainability. The UK has also subsequently moved from a net exporter of OSR oil to an importer, taking from countries that continue to use neonicotinoids, and increasing imports of palm oil which fail to address environmental goals. The regulation on neonicotinoids has therefore had profound impacts. Limited data are available, or have been sought, to ascertain if the regulation has improved pollinator health in the UK or EU.

Notwithstanding the impact observed on OSR, the extension of the ban on neonicotinoids to non‐flowering crops in 2018 was seen as presenting a risk to sugar beet crops due to losses caused by aphid‐vectored virus yellows. Accordingly, in 2020 a virus yellows incidence of 38% was observed, causing a loss of 25% of the UK crop. The severity of this loss motivated a risk‐based derogation on the use of the seed dressings which was afforded to the industry by Defra.^28^ The risk assessment used data on predicted flights of aphids provided by Rothamsted's Insect Survey; it should be noted that this derogation was not granted for use in 2025. It remains an open question as to whether sugar beet can be a viable crop in the UK without effective control of virus yellows, or if, like the case of OSR, the coping strategies of farmers will dictate a switch to another crop, leading to the need for imported sugar.

### 6.3. Weeds and herbicide resistance: blackgrass resistance characterisation and management

Rothamsted is working with farmers to build evidence on the impact of farm practices on the severity of blackgrass (*Alopecurus myosuroides*) infestation and resistance^29^ alongside understanding newly evolving resistance mechanisms. These studies have shown that field resistance is attributable to herbicide‐targeted and non‐targeted genes, adding complexity to RM and the development of diagnostics.^30^ Ongoing research is centred on the development of diagnostics tools to monitor the resistance spread in the field and association with agronomic practices. Building on this knowledge, advice to farmers can help manage both existing weed infestations and mitigate risk of further spread including introductions of newly evolved resistant biotypes. More long‐term research is centred on advanced genome analyses that set the groundwork for a better understanding of resistance in blackgrass and its close relative *Alopecurus aequalis* ^31^ and the opportunity to manipulate genetic factors. Such data can support the HRM programmes proposed by HRAC.

### 6.4. Fungi and fungicide resistance: emergent Phoma stem canker biotype on oil seed rape

A recent Rothamsted study identified, for the first time, decreased azole sensitivity in western European populations of the economically important OSR, Phoma stem canker pathogen *Plenodomus lingam*.^32^ Combined with research on molecular diagnostics development, aerial spore trapping and risk forecasting, prompt identification of resistance to specific MoA chemistries can help guide crop protection decisions, including when and what to spray, such monitoring can be used to underpin FRM strategies as proposed by FRAC to prevent or slow the emergence and spread of fungicide resistance in pathogen populations under selection by fungicide use.

#### 7. SUMMARY AND ‘CALL TO ACTION’

The unified perspectives of IRAC, HRAC, and FRAC underscore a critical challenge to global food production: the rapid erosion of our ability to manage pesticide resistance in insects, weeds and crop diseases. The primary driver is the shrinking toolbox of chemical MoAs, which are being removed from the market far faster than they can be replaced. This trend jeopardises even the most advanced IPM programmes and threatens the economic viability of farming and even competitiveness *versus* other producing regions. To avert this escalating situation, we call for immediate and concerted action to:

- Re‐evaluate Regulatory Frameworks: We urge policymakers to ensure that the withdrawal of existing crop protection tools is based on a comprehensive risk/benefit analysis that accounts for the severe consequences of losing MoA diversity for RM and overall agricultural sustainability.
- Prioritise and Accelerate Innovation: The process for evaluating and approving new, effective MoAs must be streamlined and supported to replenish the toolbox, providing growers with the rotation partners essential for RM and long‐term control.
- Strengthen Collaborative IPM: We must foster greater collaboration between regulators, researchers, industry and growers to develop and implement robust, field‐level IPM strategies that integrate all available tools – chemical, biological and cultural – to ensure their longevity and effectiveness.

Failure to act on these points will only lead to greater resistance, increased crop losses and a growing threat to food security. The time to build a more resilient and sustainable future for agriculture is now.

## Data Availability

Data sharing not applicable to this article as no datasets were generated or analysed during the current study.
